# *Bacillus* strains from Tunisian Sabkhas as promising biocontrol agents for several plant diseases in the Mediterranean

**DOI:** 10.1186/s12866-026-04819-w

**Published:** 2026-03-25

**Authors:** Haifa Ben Gharsa, Meriam Bouri, Christina Schuster, Ali Rhouma, Andreas Leclerque

**Affiliations:** 1 Department of Biology, Technische Universität Darmstadt, Schnittspahnstraße 10, Darmstadt, 64287 Germany; 2 Laboratory of Protection and Improvement of Genetic Resources of Olive, Olive Tree Institute, Tunis, Tunisia; 3 Department of Genetics and Bioengineering, Faculty of Engineering, Yeditepe University, Istanbul, Turkey; 4 Research Department, IRESA-Ministry of Agriculture, Tunis, Tunisia

**Keywords:** Sustainable development, Ecofriendly, Antimicrobial activity, Molecular characterization, Crown gall, Olive leaf spot, Blue mold

## Abstract

**Background:**

Mediterranean regions are strongly affected by many plant diseases that are responsible for serious economic losses. In respect of sustainable development, agriculture has to give equal respect to environmental, social, and profitability issues. Therefore, the management of plant diseases requires more ecofriendly solutions than conventional chemical treatments. Among new alternatives, various bacterial strains have been described as efficient biocontrol agents of plant diseases. Special importance has been accorded to saline environments as potential sources of antimicrobial activities. In this study, *Bacillus* strains were isolated from four different Sabkhas ecosystems in Tunisia and screened in vitro for their antimicrobial activities toward twelve phytopathogens.

**Results:**

Four strains, designated JS7, RS6, GO20, and ZO4,were identified as promising biocontrol candidates. The antimicrobial activity of JS7 and GO20 supernatants was more thermostable than that of RS6 and ZO4. Molecular characterization of the antimicrobial activity has shown that all strains host the genes involved in the biosynthesis of the polypeptides iturine, bacillomycin, surfactin, fengycin and plipastatin, the polyketides macrolactin, bacillaene and difficidin, and the dipeptide bacilysin. Genes involved in the biosynthesis of the bacteriocins subtilin and ericin have been detected only in strains RS6 and GO20, which belong to a separate group of rhizogenic *B. velezensis*as compared to strains JS7 and ZO4 (telluric *B. velezensis*) according to the 16S rRNA encoding sequence and housekeeping genes *purH, groEL, gyrA*and*rpoB*. JS7 and RS6 have displayed best efficacy in reducing crown gall on almond and olive leaf spot diseases in vivo. GO20 was the best in inhibiting the development of blue mold postharvest disease on apple during storage.

**Conclusions:**

Our findings highlight the importance of Sabkhas and particularly rhizospheres of halophytes as sources of antimicrobial activities and promising biocontrol agents of plant diseases for a sustainable Mediterranean agriculture.

**Supplementary Information:**

The online version contains supplementary material available at 10.1186/s12866-026-04819-w.

## Background

Plant diseases have deep impacts on global economy, ecosystem diversity and food security. Their devastating consequences are intensified by climate change. Global warming has been reported to have direct and indirect effects on plant health and pathogen outbreaks [10]. The Food and Agriculture Organization of the United Nations (FAO) has reported US$220 billion of annual global losses in crop yield caused by pathogens and pests [22]. Agriculture in Mediterranean regions is particularly experiencing phytosanitary issues related to climate change. Many studies have been carried out to predict the potential distribution and future outbreak areas under different climate change scenarios [29, 31]. For instance, isothermality and seasonal distributions of precipitations were found to be the most important climatic drivers of *Verticillium dahliae* occurrence in Mediterranean olive-growing areas of southern Spain [53]. *Spiloceaoleagina,*the anamorph of *Venturia oleaginea*(Castagne) Rossman & Crous [57]*,* is another feared fungal pathogen of olive in the Mediterranean countries. It is responsible for Peacock’s eye called also olive leaf spot (OLS) infection andcan cause serious yield losses that may exceed 20% depending on climatic conditions [36]. Although *Xylella fastidiosa* has been recently considered as the most destructive bacterial disease threatening the entire Mediterranean basin [23] after its first discovery in 2013 in Italy [64], crown gall caused by pathogenic agrobacteria is still the predominant disease encountered in the Mediterranean region, with periodic recurrences reducing the yields of marketable rootstocks [11, 25]. Under the umbrella of agricultural sustainable development, and with respect to food security, consumers health and ecosystem protection, current legislations aim to limit chemical uses for the control of phytopathogens and pests. Therefore, alternative plant disease management strategies are being actively developed to produce high quality and safe crops as well as sustainable management of agro-ecosystems. The use of biological tools such as microbe-based solutions, plant extracts and pheromone traps, is one of the most adopted practices in sustainable agriculture for the management of plant diseases and pests. Beneficial bacteria stand among the most popular biocontrol agents used in organic agriculture or in integrated pest management strategies to protect plant health and/or enhance crop production.

Several bacteria belonging to the genus *Bacillus* have been described as biocontrol agents and plant growth promoters [48, 54]. Both features are due to the production of a vast range of microbial compounds such as antibiotics or phytohormones [12, 15].

Lipopeptides (LPs) represent the most common class of compounds produced by *Bacillus spp*. [71]. They are mainly categorized into three distinct families based on the amino acid sequence: surfactins, fengycins, and bacillomycin [43]. Surfactins are powerful biosurfactants, with hemolytic, antiviral, anti-mycoplasma, and antibacterial activities [37, 49].

LPs iturin family, including iturin A, C, D, and E, bacillomycinF and L, bacillopeptin, and mycosubtilin [16, 33] exhibit strong antifungal activity against a wide range of yeasts but limited antibacterial activity [43, 68, 71]. In addition to their antimicrobial properties, lipopeptides are involved in root colonization as well as in the systemic stimulation of the host plant's immune system [43]. In general, lipopeptides act as biocontrol agents preventing plant diseases and inhibiting the progression of a wide variety of fungal plant pathogens. However, few reports have demonstrated their effects on bacteria [6].

Bacillaene, difficidin and macrolactin are polyketide compounds (PKs) with antibacterial and antifungal activities. These compounds play also a crucial role in inhibiting the growth of pathogenic microorganisms.The antibacterial polyketide bacillaene, for instance, that is produced by *B. velezensis* FZB42, demonstrated a modest bacteriostatic effect against the causative agent of fire blight disease, *Erwinia amylovora* [17]. Additionally, bacillaene-A, synthesized by various *Bacillus* spp., exhibited antifungal activity against *Termitomyces*fungi [70]. *B. velezensis* DR-08 secreted difficidin and oxydifficidin displayed antibacterial activity against *Ralstonia solanacearum* [28].

Efficient *Bacillus* strains used in pest management have different origins. Identifying efficient bacterial strains to control plant diseases requires efficient research strategies and screening methods. Recently, microbiota of specific habitats under harshconditions have been described to be involved in biotic and abiotic stress tolerance enhancement of crops [55]. Microbe-based biotechnology in agriculture has developed new perspectives of some extremophile applications for the enhancement of crop production through plant growth promotion (PGP) and/or biocontrol of plant pathogens [12, 45].

Saline soils have been described as great sources of beneficial networking between plants and microbes [21]. Several studies have described bacteria from rhizosphere of halophytes with antagonistic activities against several phytopathogens [4, 7]. “Sabkhas” are very specific coastal ecosystems characterized by supratidal mudflat or sandflat in which saline minerals accumulate as the result of evaporation in semiarid to arid climate [41]. Sabkhas are considered as gradational between land and intertidal zone within restricted coastal plains just above normal high-tide level [67]. The vegetation of the Sabkhas is characterized by perennial halophyte communities such as belonging to the genera *Suaeda*, *Zygophyllum*, *Tamarix*, *Cressa*, or *Salicornia* [20] as well as palm trees [2]. Saline biotopes are believed to harness a diversity of microorganisms of potential use in agriculture.This study aimed to explore the microbial diversity of saline environments by screening soils from four Tunisian Sabkhas for *Bacillus* strains with biocontrol potential. The objective was to identify and characterize efficient strains that could be used to manage major plant diseases in Mediterranean regions. Selected isolates were subjected to biochemical and molecular analyses to better understand their mechanisms of action.

## Methods

### Bacterial isolation

Bacteria were isolated from different Tunisian biotopes, either from bulk soil or plant rhizosphere (Table 1). Isolation was on Luria Bertani agar (LBA: 10 g tryptone, 5 g yeast extract, 5 g NaCl, 20 g agar in 1 L of distilled water and a pH adjusted at 7.2 before sterilization). Ten grams from each bulk soil sample were added to 90 ml of sterile distilled water in flasks. For rhizobacteria isolation, plants roots were gently washed twice in sterile distilled water to remove adhering soil fragments. Ten grams of rhizospheric soil were added to 90 ml of sterile distilled water (SDW) and shaken at 200 rpm for 30 min. Then serial dilutions were spread on LBA and incubated at 25 °C for 72 h. Bacteria from emerging colonies were preliminarily characterized by Gram staining and purified on LBA; pure cultures were stored in 30% glycerol at −20 °C.

**Table 1:** Origins of isolated bacteria in this study

Isolate	Region	Geographic coordinates	Origin of isolation
RS6	El Ataya, Kerkennah, Sfax	34°73′35,8’’N 11°29′80,5’’E	Rhizosphere of *Zigophyllum album*
JS7	Sebkhet de Sidi El Heni, El Djem, Sousse	34°44′02.5"N 11°18′07.1"E	Soil
GO20	Chott elFejej, Rejim Maatoug, Gabes	33°27′26.3"N 8°19′35.6"E	Rhizosphere of *Phoenix dactylifera L*
ZO4	Chott elFejej, Rejim Maatoug, Gabes	33°26′55.7"N 8°19′20.8"E	Soil

### Screening of antimicrobial activity in vitro

The antimicrobial activity of the bacterial isolates towards several phytopathogenic bacterial and fungal species (Table 2) was determined by double layer and agar well diffusion methods as described by Rhouma et al. [54] with some modifications. Both methods were performed in three replicates for each representative tests.

**Table 2:** Bacterial and fungal reference strains used in this study

Phytopathogenic species	Source	Growth Medium (*)	Incubation conditions
*Agrobacterium fabrum*C58	Collection of Microbial Ecology, University of Lyon, France	MG	26 ± 1 °C
*A. vitis*(CFBP5523)	Collection of Microbial Ecology, University of Lyon, France	MG	26 ± 1 °C
*A. tumefaciens* B6	Collection of Microbial Ecology, University of Lyon, France	MG	26 ± 1 °C
*Xanthomonas* *juglandis* (1325.2b)	Collection of InstitutValenciàd'InvestigacionsAgràries (IVIA), Valencia, Spain	LB	30 °C
*X. campestris* (339.6)	Collection of InstitutValenciàd'InvestigacionsAgràries (IVIA)	LB	30 °C
*Pectobacteriumcarotovorum*(1001)	Collection of InstitutValenciàd'InvestigacionsAgràries (IVIA)	LB	30 °C
*Verticillium dahliae* V4	Collection of Olive Institute- Tunis, Tunisia	Potato Dextrose Agar (PDA)	30 °C
*Rhizoctonia bataticola*HQ392809	Collection of Olive Institute- Tunis, Tunisia	PDA	30 °C
*Fusarium oxysporum*JN400698.1	Collection of Olive Institute- Tunis, Tunisia	PDA	30 °C
*Fusarium solani*FJ874633	Collection of Olive Institute- Tunis, Tunisia	PDA	30 °C
*Penicillium expansum*	Collection ofCenter of Biotechnology of BorjCédria (CBBC), Tunisia	PDA	28 ± 2 °C
Conidia of*Spilocaeaoleagina*	This study	Olive leaf extract (OLE) [59]	20 ± 2 °C

(*) MG: L-Glutamicacid (2 g/l), Mannitol (10 g/l), KH_2_PO_4_ (0.5 g/l), NaCl (0.2 g/l), MgSO_4_.7H_2_O (0.2 g/l), Agar (20 g/l); LB: tryptone (10 g/l), yeast extrat (5 g/l), Na Cl (5 g/l), agar (20 g/l); PDA: (Merck, Germany). OLE (according toSaad and Masri, 1978): glucose (5 g/l), olive leaf extract (20 g of health fresh leaves/l), agar (15 g/l). All media’s pH were adjusted at 7.2 before sterilization

#### Double layer method (DLM)

A 20 µl spot of bacterial suspension of isolates (10^7^ CFU ml^−1^) was inoculated on LB medium. After incubation at 25 °C for 2 days, the grown bacterial colonies were exposed to chloroform vapor for 30 min then left open for 15 min in a laminar flow cabinet. A one milliliter suspension of phytopathogenic microorganisms (10^7^ CFU ml^−1^ and 10^4^ spores ml^−1^ of bacteria and fungi, respectively) was mixed with 3 ml of LBA (0.6% agar) at 45 °C and was quickly inoculated by spreading on the plates containing the potentially antagonists. Plates were incubated at 25 °C and checked after 24 to 48 h for the appearance of inhibition zones surrounding the bacterial spots. The experiment was repeated three times.

#### Agar well diffusion method (AWD)

Antagonistic bacteria revealed according to the double layer method were transferred individually to 50 ml of Luria Bertani broth medium (LB broth) in a 250 ml Erlenmeyer flask and incubated by shaking at 200 rpm for 4 days at room temperature. One milliliter of phytopathogenic microorganisms’ suspension (10^7^ CFU ml^−1^ and 10^4^ spores ml^−1^ for bacterial and fungal agents, respectively) was mixed with 3 ml of their corresponding agar growth media (0.6% agar) at 45 °C and quickly spread on plates containing LBA medium, in which 4 wells of 6 mm diameter will be punched after solidification of the medium. The antagonist cultures were centrifuged at 15,000 rpm for 30 min and microfiltred (22 µm), then 100 µl of each sample were filled into the wells. Plates were incubated at 25 °C and were subsequently examined for inhibition zones around wells for size recording. Three separate tests were made.

#### Dual-culture assay for fungi (DCA)

*Bacillus* isolates were tested for antagonistic activity against pathogenic fungi by dual-culture assay as described by Sakthivel and Gnanamanickam [62]. Briefly, bacterial isolates were streaked in the middle of a PDA plate. A fungal disc of 6 mm diameter (from a 72 h a PDA culture) was inoculated at an equal distance from the bacteria and the periphery of the other side of the petri dish. Plates inoculated with the fungal discs and SDW were used as negative control. The plates were incubated at 30 °C for 3–5 days. The antagonistic effect of *Bacillus* isolates was expressed as the percentage of inhibition of the fungal growth by using the following equation [40]:

$$\text{Inhibition of radial growth }\left(\mathrm{\%}\right)=\left[\left(\mathrm{R}1-\mathrm{R}2\right)/\mathrm{R}1\right]\times 100,\text{ where}:$$

- R1: diameter of pathogen growth from the other side of the dish.
- R2: diameter of pathogen growth from the side of the bacteria.

### Enzymatic activities

#### Proteinase activity

The capacity of the bacterial strains to degrade protein was identified by halos forming after 48 h of strains incubation in GNL medium (pastone 5 g/l, yeast extrat 3 g/l, agar 18 g/l, 250 ml of sterile skimmed milk, 750 ml of SDW and pH 9).

The proteinase activity was measured according to the method of [30]. Briefly, it consists of mixing 0.5 ml of a casein solution 1% (w/v) in a pH 9 tampon solution, with 0.5 ml of the beforehand diluted bacterial supernatant solution. After 15 min of incubation at 60 °C, the reaction was stopped by the addition of 0.5 ml trichloroacetic acid solution (TCA) 20% (w/v), which acts to precipitate the casein not hydrolyzed. Fifteen min later, this reactor medium was centrifuged during 15 min on 13000 rpm/min. Finally, the absorbance of the bacterial supernatant was measured at OD_280_ nm. A unit of proteinase activity (AP) is defined as the quantity of enzyme released from 1µg of tyrosine by ml and by min. A curve of calibration (0–100 µg/ml) of tyrosine is realized in the same conditions.

#### Chitinase activity

The chitinase activity was measured in the supernatant of 72 h incubated strains in liquid LB medium, according to the colorimetric DNS method of Miller [39] as the following: 0.4 ml a colloidal chitin solution 0.5% (w/v) is mixed in a pH9 tampon with 0.4 ml of a bacterial supernatant. After 5 min of incubation at 55 °C, the mixture is centrifuged for 10 min at 13,000 rpm. Then, 0.5 ml of the supernatant is added to 0.5 ml of DNS. This new reactor medium is incubated during 10 min at 100 °C. After cooling, the absorption of the supernatant is measured at OD 550 nm. The values are converted to an enzymatic activity based on a calibration curve (0–100 µg/ml) N-acetyl glucosamine (NAG),realized in the same conditions. One unit of a chitinase activity is defined as the quantity of enzyme released from 1µg of NAG by ml by min.

### Preliminary characterization of antibacterial compounds

The characterization of the antibacterial compounds of the most efficient bacterial strains was achieved using supernatant samples collected from 72 h cultures.

#### Proteinase sensitivity

Sensitivity of antibacterial compounds to proteolytic enzymes (proteinase K) was determined by incubation of cell-free supernatant samples for 1 h at 37 °C with proteinase K (1 mg/ml). After incubation, proteinase K was heat inactivated for 3 min at 100 °C.

#### Thermal stability

Thermal stability of antibacterial activity was determined by incubation of aliquots (500 µl) of cell-free supernatant at 40 °C, 60 °C, 80 °C and 100 °C for 30 min.

#### pH sensitivity

The activity of the antibacterial compound at different pH values was estimated after storage of the supernatant culture for 1 day at 4 °C in buffer ranging from pH 3 to pH 9. After incubation at indicated pH, the medium was neutralized before the assay was conducted.

#### Molecular characterization

Several polymerase chain reactions (PCR) were performed to determine the presence of antimicrobial genes responsible for the biosynthesis of subtilin, subtilosin, ericin, sublancin, iturin, mycosubtilin/iturin, surfactin/lichenysin, fengycin, plipastatin, bacillomycin, difficidin, bacillus, macrolactin and bacilycin biosynthesis genes in the DNA of all strains.Primers used are listed in the Suppl. Table 1.PCR amplifications were carried out using the Taq PCR Kit (NEB). The PCR conditions are as follows: an initial denaturation step of 2 min at 95 °C, followed by 35 cycles of 45 s at 95 °C, 45 s at 52 °C, 2 min at 68 °C then a step final elongation of 5 min at 68 °C.

### Genetic diversity

The molecular taxonomic characterization of the bacterial isolates was conducted as previously described [7]. The isolates were cultivated in liquid LB medium for 24 h at 28 °C and 120 rpm. Genomic DNA was extracted using the DNeasy Blood & Tissue Extraction kit (Qiagen). PCR amplification of 16S*, groEL, purH, rpoB*, and *gyrA* marker genes was carried out using the Taq PCR Kit (NEB) with the following PCR program: initial denaturation at 95 °C for 5 min, 35 cycles of denaturation at 95 °C for 40 s, annealing at 55 °C for 1 min and elongation at 68 °C for 2 min, and final elongation at 68 °C for 4 min. The sequences of the primers are listed in Suppl. Table 1. After verifying amplification through 1% horizontal agarose gel electrophoresis, PCR products were purified using the Qiaquick PCR Purification kit (Qiagen) and sequenced by an external provider (Microsynth AG, Gießen, Germany). Consensus sequences were generated from raw sequence data using the MEGA 6 software tool [66], and phylogenetic reconstructions employed the Neighbor Joining (NJ) method [61].

### Biocontrol assays in vivo

Different plant models and their corresponding phytopathogens were tested as described in Table 3.

**Table 3:** Details of biocontrol assays in vivo

Plant models	Plant diseases (phytopathogens)	Methods	References
Almond (*Prunus dulcis* variety *dulcis*)	Crown gall (*Agrobacterium fabrum*C58)	Stem inoculation	[7]
Apple (*Malus domestica*) Golden Delicious variety	Blue Mold of apples (*Penicillium expansum*)	Postharvest Fruit inoculation	[32] with modifications^(**a**)^
Tomato (*Solanumlycopersicum* cv. Rio Grande)	Verticillium wilt of tomato (*Verticillium dahliae*)	Root-dip method	[24]
Olive tree (*Olea europaea* var Chemlali)	Olive scab or leaf spot (OLS) (*Spilocaeaoleagina*)	• Spore Germination assay on detached olive leaves • Aerial part treatment in field	• Salman, 2017 with modifications^(**b**)^ • This study^(**c**)^

- Biocontrol of blue mold of apples: Apples were superficially disinfected by immersion in a sodium hypochlorite solution (2% active chlorine) for 1 min, before to be rinsed twice with sterile distilled water (SDW) and dried at room temperature. One wound was performed from the side in the middle of the fruit with a sterile needle (Set Medikal, Turkey). Wounds were treated with 30 µl of *Bacillus* cell suspension at a concentration of 10^8^ cfu/ml in SDW. Negative control group was treated with 30 µl of SDW. After 2 h at room temperature each wound was inoculated with 15 µl of *P.expansum* spore suspension (10^6^ spores/ml). Fruits were incubated in the dark at 24 °C for 7 days with 85% RH. Three fruits were used for each treatment and the experiment was performed three times.
- OLS spore germination assay: One hundred of olive leaves with obvious symptoms of OLS from the region of Morneg (North of Tunisa), were cut into 5 cm^2^ segments, and shaken in DSW at 100 rpm for 30 min to check the conidial viability of *S. oleagina*. One hundred microliter of the conidial suspension adjusted to 10^4^ conidia/ml were plated on an olive leaf extract agar medium (OLE) prepared as described by Saad and Masri [59]. After 48 h of incubation at 20 °C in dark, the percentage of germination was recorded on double-sided adhesive paper after lacto-fuchsin staining as described by Benitez et al. [8]. The germination was considered positive if the length of the germ tube exceeded half of the conidial length [42].

The effect of *Bacillus* strains on the germination of *S. oleagina* conidia was recorded as described by Salman [63] with modifications. Briefly, 100 μl of antagonistic bacterial suspension (10^8^ cfu/ml) and 100 μl of fungal conidia (10^6^ conidia/ml) (prepared from infected olive leaves showing at least 90% of *S. oleagina* viable conidia as described above) were mixed with 800 μl of sterile Phosphate Buffered Saline (1X) (PBS) (Sigma, UK) for 15 min at 100 rpm. Solution of Bordeaux mixture (BM) 1% and DSW were used as positive and negative control, respectively. Twenty microliters of the mixture suspension were dropped in 5 replicates on slides of OLE medium and incubated at 20 °C, RH ≥ 85 and dark for 24 h. The germination was recorded as described above and the inhibition rate was calculated from 100 randomly selected conidia in each droplet as follows:

Germination inhibition rate = [(C-T)/C] × 100 where C and T are the conidial germination values in negative control and treated plates, respectively. The trial was repeated three times.

(iii) OLS in field biocontrol assay: The experiment was carried in olive orchards (variety of Chetoui situated in the region of Morneg, Tunisia). The infection of OLS disease (*S. oleagina*) was estimated to be more than 80% (according to symptoms observed on leaves). Arial parts of olive trees were treated by 2 L of bacterial suspension (10^6^ CFU/ml) using a low-pressure sprayer. BM (1%) and SDW were used as positive and negative control, respectively. The experimental design was completely randomized. Five olive trees was used by treatment and the trial was repeated twice (springs of 2014 and 2016). After 60 days, 100 leaves were collected from the four sides (North, West, South, and East) of treated trees. Leaves were transported to the laboratory at 4 °C to be checked for typical OLS symptoms.

### Statistical analysis

ANOVA tests of variance were performed using SPSS software (version20) for statistical analyses. The Duncan’s test was applied to determine significance of mean differences with a level of *P* = 0.05 for antimicrobial activity in vitro, proteinase and chitinase activities.

Bootstrapping statistics for phylogenetic tree topologies were carried out using the MEGA 6 software package.

## Results

### Bacillus isolation

A total of 192 isolates were recovered on LB agar from four different Sabkhas biotopes (Table 1) known to be under saline and drought stresses. Sixty-six putative *Bacillus*isolates were selected according to their typical Gram-positive rod-shape microscopic morphology after staining.

### Antimicrobial activity in vitro

All 66 putative*Bacillus* isolates were screened for their antagonistic activity using the DLM and DCA first for phytopathogenic bacteria and fungi, respectively (Tables 4 and 5). One strain from each biotope was identified as potential biocontrol agent according to the diameters of inhibition zones of the in vitro assays and (Supp. Table 2), therefore, selected for downstream analyses (Table 1). In case of an inhibition activity, the antimicrobial effect of their supernatant was assessed according to AWD method (Tables 4 and 5).

**Table 4:** Diameters of inhibition zones (mm) obtained by antagonistic*Bacillus* strains against phytopathogenic bacteria in double layer culture and agar well diffusion assays

Double Layer Method (DLM)						
	*A. tumefaciens* C58	*A. vitis* (CFBP5523)		*X. campestrispv. juglandis* (1325.2b)	*X. campestris* (339.6)	*P. carotovorum* (1001)
JS7	21.03 ± 1.66a	12.87 ± 1.37a		15.24 ± 1.43a	9.56 ± 0.42a	6.58 ± 1.24a
RS6	20.13 ± 1.23a	13.36 ± 1.62a		8.89 ± 0.56b	8.89 ± 0.56a	5.61 + 0.31a
GO20	12.20 ± 1.11b	0b		12.66 ± 1.69c	0b	0b
ZO4	16.34 ± 1.08c	0b		9.90 ± 0.19c	0b	6.51 ± 1.1a

Agar Well Diffusion Method (AWD)						
	*A. tumefaciens* C58		*A. vitis* (CFBP5523)	*X. campestris* (339.6)	*X. campestrispv.juglandis* (1325.2b)	*P. carotovorum* (1001)
JS7	22.96 ± 0.24a		13.52 ± 0.27a	18.03 ± 1.35a	9.98 ± 0.42a	6.7 ± 0.35a
RS6	19.58 ± 0.15b		11.92 ± 0.68b	11.12 ± 0.4c	8.23 ± 0.6a	5.64 ± 0.17a
GO20	12.03 ± 0.08c		NT	13.36 ± 0.56b	NT	NT
ZO4	17.65 ± 0.37b		NT	10.13 ± 0.41c	NT	6.42 ± 0.15a

Values are mean ± standard deviation (*n* = 3). Within each column, values followed by the same lowercase letter are not significantly different according to Duncan’s multiple range test (*p* = 0.05)

*NT* Not testedin AWD assays due to absence of activity in initial DLM screenings

**Table 5:** Percentage of inhibition (%) and diameters of inhibition zones (mm) of phytopathogenic fungi by *Bacillus* isolates according to dual-culture assay and Agar well diffusion Method, respectively

Dual Culture Assay (DCA) (inhibition in %)					
	*Verticillium dahliae* V4	*Rhizoctonia bataticola*	*Fusarium solani*	*Fusarium oxysporum*	*Penicillium expansum*
JS7	68.17 ± 0.66a	33.12 ± 0.95a	61.17 ± 0.64b	46.67 ± 0.31a	48.50 ± 0.41b
RS6	56.87 ± 1.52c	22.12 ± 0.74c	66.13 ± 0.47ab	41.77 ± 0.14b	57.38 ± 0.33a
GO20	62.88 ± 1.66b	12.96 ± 0.66d	70.18 ± 0.61a	42.16 ± 0.70b	58.33 ± 0.26a
ZO4	61.63 ± 0.77b	32.61 ± 0.73b	52.61 ± 0.64c	43.18 ± 0.82ab	0c

Agar Well Diffusion Method (AWD) (inhibition in mm)					
	*Verticillium dahliae* V4	*Rhizoctonia bataticola*	*Fusarium solani*	*Fusarium oxysporum*	*Penicillium expansum*
JS7	20.44 ± 0.21b	13.36 ± 0.95a	13.59 ± 0.11b	11.76 ± 0.50a	17.34 ± 0.15b
RS6	22.68 ± 0.52a	11.87 ± 0.88ab	12.15 ± 0.16b	10.23 ± 0.33b	22.68 ± 0.8a
GO20	17.34 ± 0.39c	8.22 ± 0.41bc	16.88 ± 0.22a	8.52 ± 0.51c	23.12 ± 0.25a
ZO4	17.76 ± 0.21c	6.26 ± 1.02c	11.42 ± 0.18b	11.48 ± 0.27a	NT

Values are mean ± standard deviation (*n* = 3). Within each column, values followed by the same lowercase letter are not significantly different according to Duncan’s multiple range test (*p* = 0.05)

*NT* Not tested in AWD assays due to absence of activity in initial DLM screenings

**Fig. 1:**
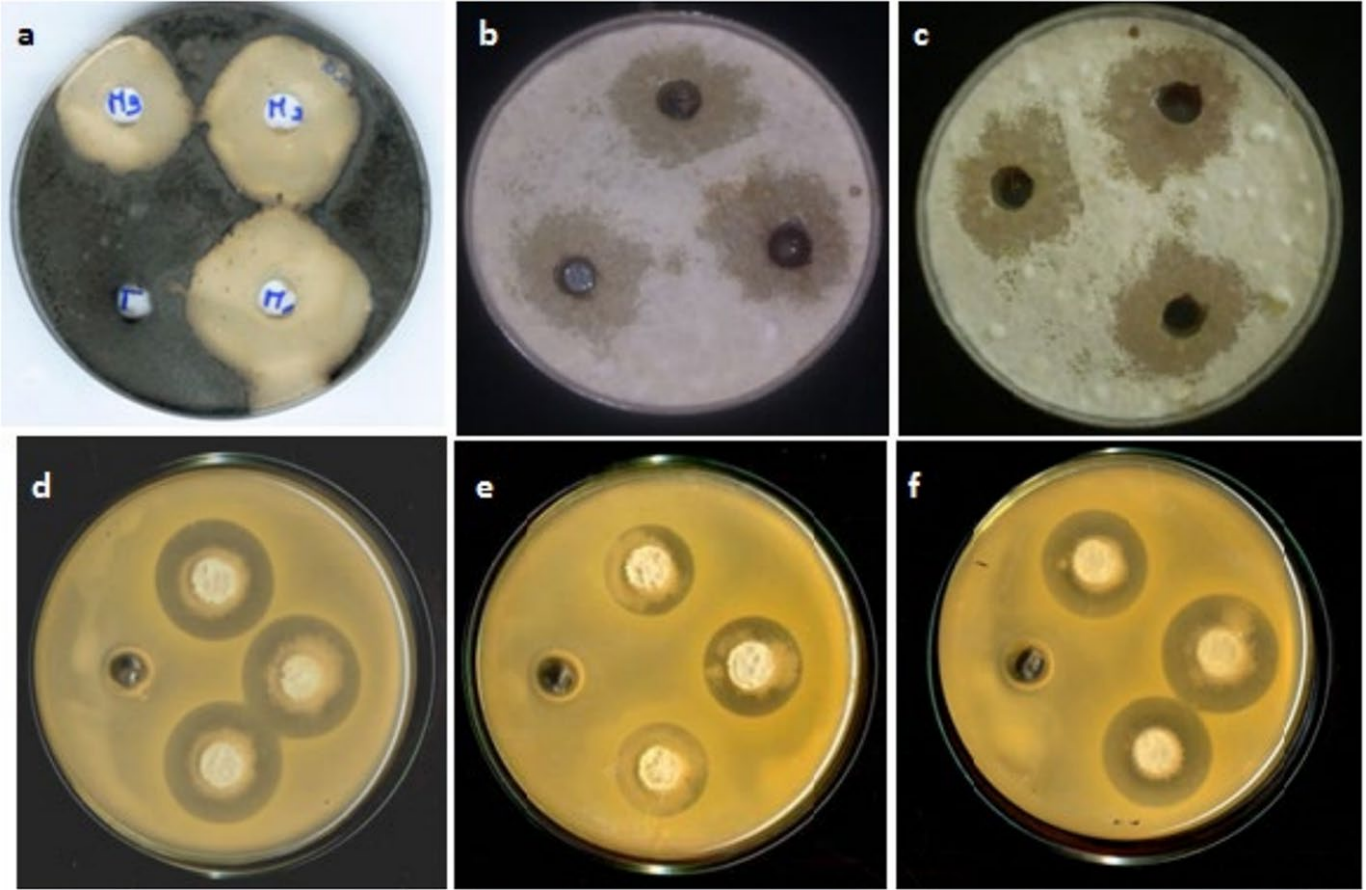
Antimicrobial activity of some Bacillus isolates against main phytopathogens: (**a**) JS7 vs R. bataticola, (**b**) RS6 vs F. oxysporum, (**c**) ZO4 vs F. solani, (**d**) GO20 vs A. tumefaciens, (**e**) JS7 vs P. carotovorum, (**f**) ZO4 vs X. campestris

According to the statistical analysis of inhibition zones diameters, the best antimicrobial activities were recorded with (JS7 and RS6) *vs A. tumefaciens*; (RS6 and JS7) *vs A. vitis*; JS7 *vsX. juglandis*, (JS7 and RS6) *vsX. campestris* and (JS7 and ZO4) vs *P. carotovorum *(Fig. 1e and f). Isolates JS7 and RS6 showed broadest antibacterial spectrum. GO20 showed more specific activity towards phytopathogenic bacteria. No substantial differences in inhibition zone diameters were recorded between the two methods DLM and DCA.

Except isolate GO20, *Bacillus* strains exhibited antagonistic activities against all phytopathogenic fungitested. In contrast to antibacterial assays, the difference between inhibition zonediameters obtainedfor the same strain byboth methods employed, i.e. DCA and AWD, was statistically significant in antifungal assays. Statistical analysis of inhibition percentages recorded with DCA revealed best antifungal activities for the following antagonist vs pathogen combinations: JS7 *vsV. dahliae*; (JS7, and ZO4) *vs R. bataticola*;GO20 *vsF. solani*; JS7 *vsF. oxysporum* and (RS6 and GO20) *vs P. expansum*.However, according to AWD assay the best antagonistic activities were recorded with: RS6 *vs V. dahliae*; JS7 *vsR. bataticola*; (GO20) *vs F. solani*; (JS7, ZO4 and RS6) vs. *F. oxysporum* and (GO20 and RS6) *vsP. Expansum *(Fig. 1a, b and c).

### Proteinase and chitinase activities

**Fig. 2:**
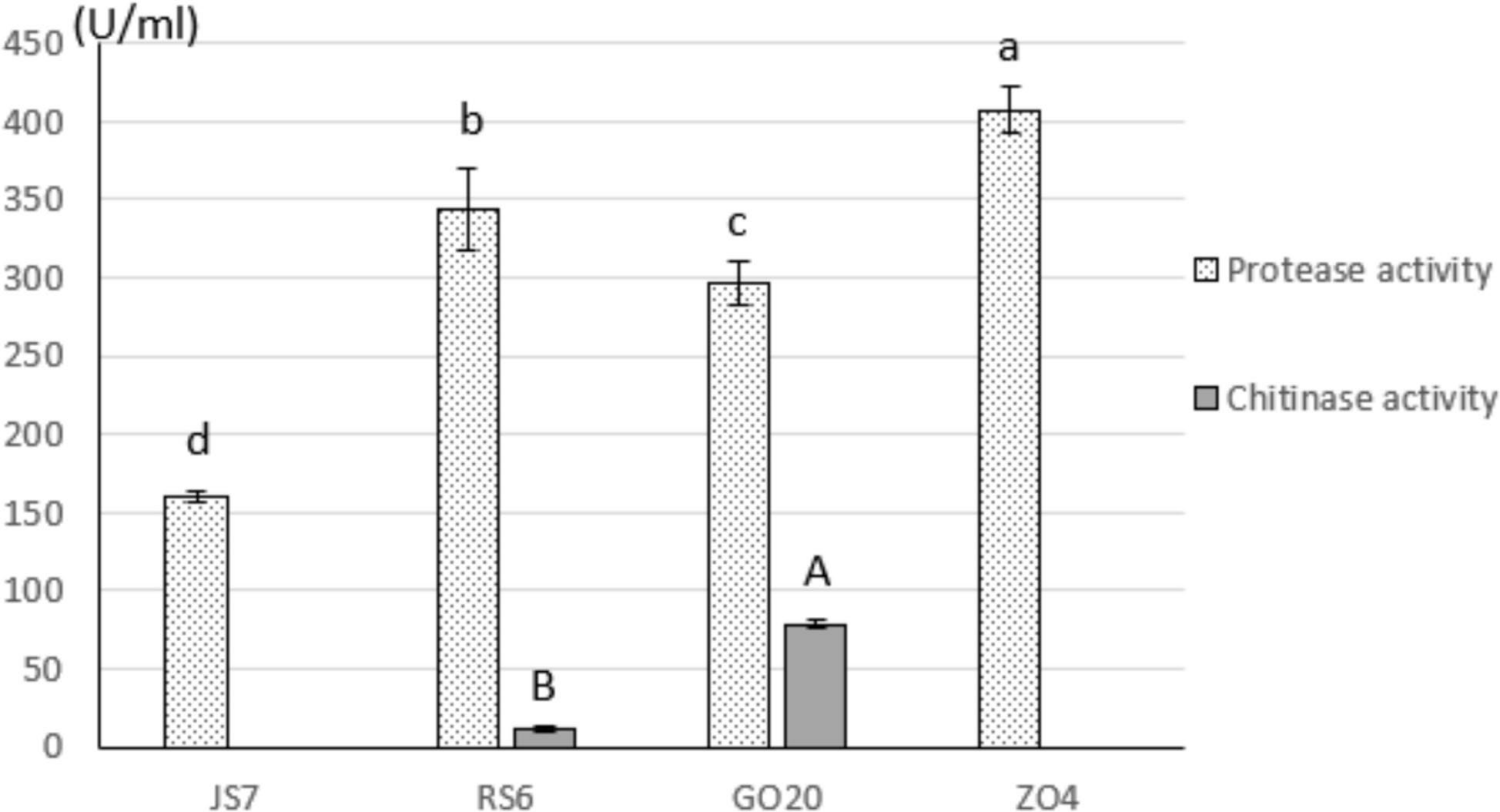
Amounts of protein and chitin degradation by different *Bacillus* isolates. Error bars represent standard deviation (*n* = 3). Lowercase and uppercase letters designate homogenous group based on Duncan’s multiple range test for protease and chitinase activity, respectively (*p* = 0.05)

Proteinase activity was observed on GNL medium with the degradation of casein by all four*Bacillus* isolates (Fig. 2). However, chitinase activity was recorded with two isolates only. The best amounts of protein and chitin degradation were obtained with ZO4 (407.27 U/ml) and GO20 (78.37 U/ml), respectively (Fig. 2).

### Preliminary characterization of antibacterial compounds

**Fig. 3:**
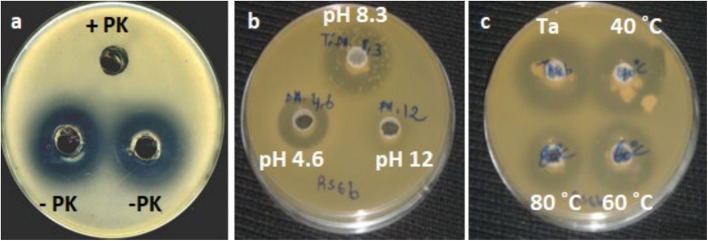
Sensitivity of the antimicrobial activity of RS6 supernatant against A. tumefaciensC58 after treatment with: (**a**) supernatant activity before (-PK) and after (+PK) proteinase K treatment, (**b**) different pH values (pH=4.6; 8.3 and 12), (**c**) different temperatures (Ta: ambient temperature, 40˚C, 60˚C and 80˚C)

**Fig. 4:**
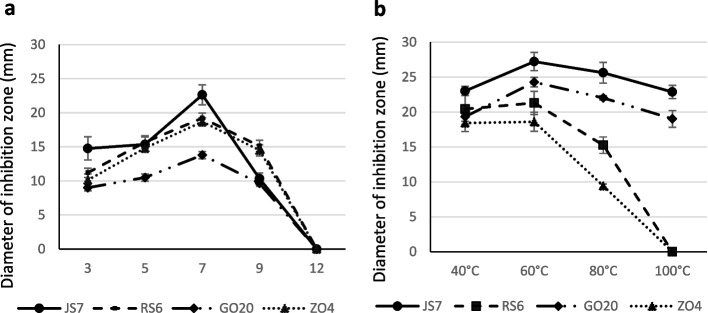
Stability of the antimicrobial activity of *Bacillus* supernatants at different pH (**a**) and temperatures **b**. Error bars represent standard deviation (*n* = 3)

All bacterial supernatants tested lost their antimicrobial capacity against representative phytopathogenic bacteria and fungi after their treatment with proteinase K (Fig. 3), which shows the proteinaceous nature of the antimicrobial compounds.At pH 7, the supernatant of all strains showed an optimal antimicrobial activity which was remarkably reduced at pH 9 and completely lost at pH 12 (Fig. 4). Interestingly, strains JS7 and GO20 retained greater antibacterial activity after thermal treatment at 80 °C and 100 °C compared to RS6 and ZO4, suggesting higher thermostability of the bioactive compounds.

#### Screening for antimicrobial genes

Detection of components involved in the antagonistic activity of different strains by PCR showed that for all strains their genomes do not contain the genes involved in the biosynthesis of bacteriocins, sublancin (*sunT*), mycosubtilin (*myc/itu*) and subtilosin (*yiwB, sboA, albA*). Also, all of them host the genes involved in the biosynthesis of the lipopeptides iturine (*ituA, ituD, ituC*), bacillomycin (*bmyA*), surfactin (*srf*), fengycin (*fen*) and plipastatin (*pps*), the polyketides macrolactin (*mlnA*), bacillaene (*baeA*) and difficidin (*dfnA*) and the dipeptide bacilysin (*bacA/B*).Notably, rhizospheric strains RS6 and GO20 carried a distinct profile of bacteriocin-related genes compared to telluric isolates JS7 and ZO4. The genome of strains RS6 and GO20 contains the genes encoding for the biosynthesis of the bacteriocins subtilin (*spaS*) and ericin (*eriC, eriSa* and *eriSb*) while these genes are absent from strains JS7 and ZO4 (Table 6).

**Table 6:** PCR detection of bacteriocin, lipopeptide and polyketide biosynthesis genes (-: absence of the gene; + : presence of the gene)

	Primers	RS6	JS7	GO20	ZO4
Subtilisin	Osbo P1N/P2N	-	-	-	-
Ericin	Eric_F/R	+	-	+	-
Subtilin	SpaS_Fwd/Rev	+	-	+	-
Mycosubtilin	Am1-F/Tm1-R	-	-	-	-
Fengycin	Af2-F/Tf1-R	+	+	+	+
Plipastatin	Ap1-F/Tp1-R	+	+	+	+
Surfactin	As1-F/Ts2-R	+	+	+	+
	SRFA-F1/R1	-	-	-	-
	SFP-F1/R1	-	-	-	-
Sublancin	SUNT-F1/R1	-	-	-	-
Iturin A	ITUD1F/1R	+	+	+	+
Iturin C	ITUC-F1/R1	+	+	+	+
Iturin D	ITUD-F1/R1	+	+	+	+
Bacillaene	baeR_F/R	+	+	+	+
Macrolactin	mlnA_F/R	+	+	+	+
Bacilysin	bacA/B_F/R	+	+	+	+
Bacillomycin	bmyA_F/R	+	+	+	+
Difficidin	dfnA_F/R	+	+	+	+

### Genetic identification and diversity

In order to identify all the strains, an amplification of the 16S region and the housekeeping genes *purH, groEL, gyrA and rpoB* followed by sequencing were carried out. Phylogenetic analysis based on sequences of the 16S rRNA region was not sufficient to differentiate between species within a complex due to the highly conserved nature of this region (data not shown).Only the consensus alignment of the *groEl, rpoB, gyrA*and*purH* genes allowed to construct the phylogenetic tree by comparing with homologous nucleotide sequence data from *Bacillus* reference strains. Amplified marker sequences were submitted to the GenBank database under accession numbers PQ583503-PQ583514 and PQ623110-PQ623113.

**Fig. 5:**
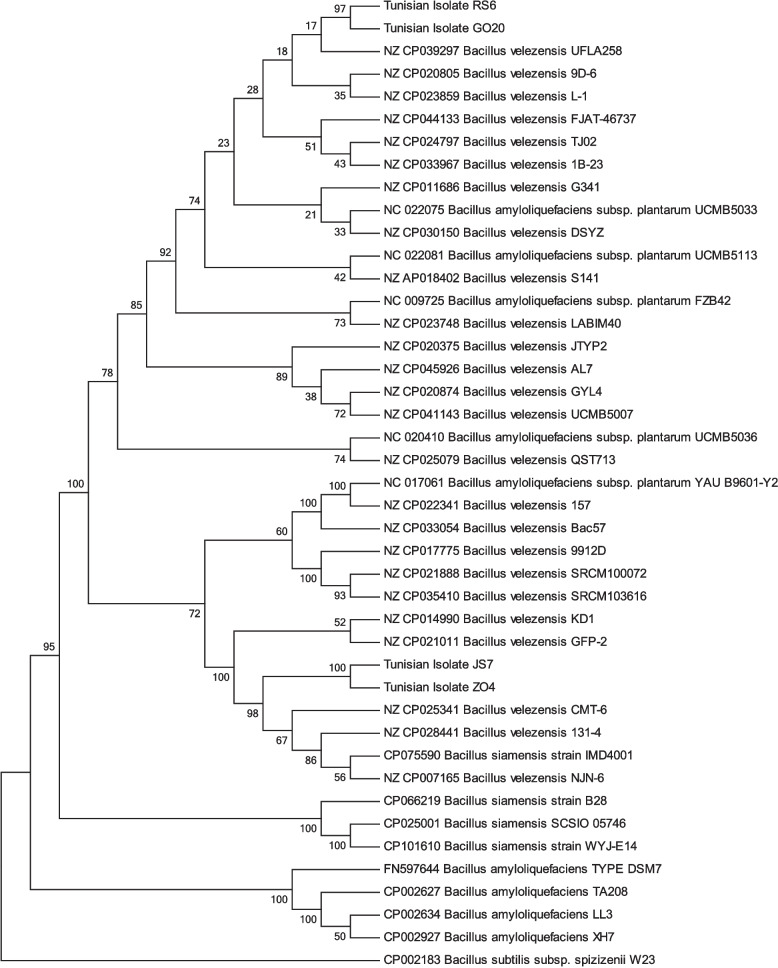
Neighbor Joining (NJ) phylogeny of bacteria belonging to the Operational Group *Bacillus amyloliquefaciens* as reconstructed from concatenated *rpoB, purH, groEL*and *gyrA.* marker sequences

The reconstruction of the phylogenetic tree by the concatenation of the four markers showed that all strains studied belong to the species Bacillus velezensis and can be assigned to two distinct groups (Fig. 5).

### Crown gall biocontrol assay

**Fig. 6:**
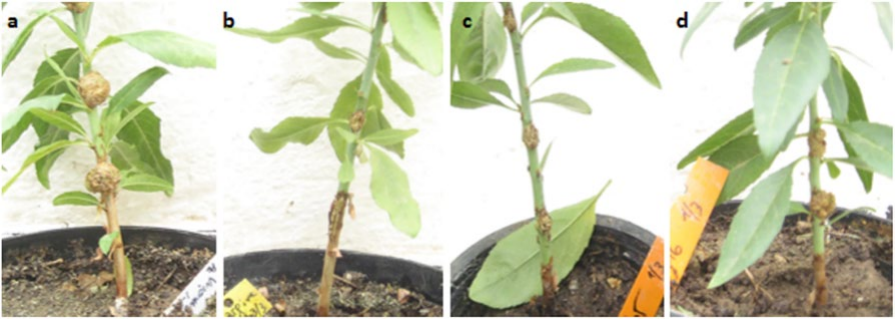
Inhibition of A. *tumefaicens* C58 tumor formation on almond stems under pre-treatment by Bacillus strains 70 days after inoculation: (**a**) DSW as Negative Control, (**b**) strain JS7, (**c**) strain RS6 and (**d**) strain GO20. For each treatment, three plants were used and for each plant three inoculation sites were performed in one representative experiment

Only three isolatesshowing best antimicrobial activities in vitro against A. tumefaciens C58 were selected for crown gall biocontrol in planta. The effect of almond stems pretreatment with JS7, RS6 and GO20 strains on tumor induction by A. tumefaciens C58, after 70 days of inoculation is illustrated in Table 7 and Fig. 6. The results revealed that Bacillus strains were effective in reducing the number and fresh weight of tumors. The number of tumor formations was reduced to 1/9 with JS7, 3/9 with RS6 and GO20 comparing to 9/9 with the negative control (DSW). The fresh weight of induced tumors was also reduced as compared to the control. The best inhibition activity was recorded with strain JS7 with only one tiny tumor (0.027 g).

**Table 7:** Effect of pretreatment by *Bacillus* strains on tumor formation induced by *A. tumefaciens* C58 in inoculated almond stems

Strains	Nr. of inoculated site	Nr. of tumors	Fresh weight of tumors (g)
DSW	9	9	0.755 ± 0.045a
JS7	9	1	0.027 ± 0.00c
RS6	9	3	0.038 ± 0.06c
GO20	9	3	0.456 ± 0.035b
ZO4	9	7	0.5960.040ab

Values are mean ± standard deviation. Lowercase letters designate homogenous group based on Duncan’s multiple range test (*p* = 0.05)

### Biocontrol of olive leaf spot (OLS)

**Fig. 7:**
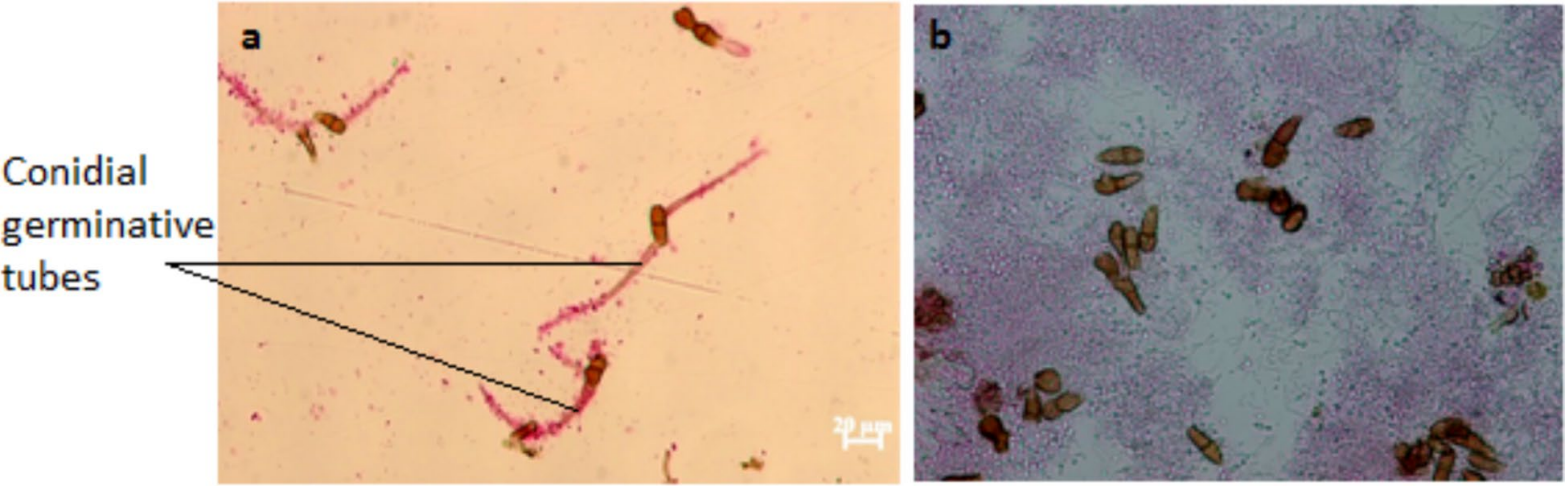
Inhibition of *S.oleagina* conidial germination in the presence of strain JS7 (**b**) as compared to the negative control treatment **a**

FourBacillus strainswere tested on infected olive leaves showing at least 90% of viable S. oleagina to check out their effect on conidial germination compared to negative and positive controls (Table 8). All strains showed inhibitory effect on the germination of S. oleagina compared to the negative control. The best antagonistic activity was recorded with strain JS7 (Fig. 7). Only 1.93% of conidial germination was recorded with JS7 comparing to 2.13% with the positive control (BM), without any statistical significance (P = 0.05). For this reason, JS7 was selected for the in vivo assay.

**Table 8:** The antagonistic effect of different *Bacillus* strains on *S. oleagina* assessed through conidial germinations and the percentage of infested leaves in orchards as compared with negative and positive controls

Treatment	Conidial germination on olive leaves (%)	Infested leaves in orchards (%)	
		Old leaves	Young leaves
Negative control (SDW)	94.47 ± 3.35^a^	98.20 ± 16.16^a^	64.20 ± 9.23^a^
Positive control (BM)	2.13 ± 0.52^c^	95.10 ± 7.81^a^	21.40 ± 5,91^b^
JS7	1.93 ± 0.51^ cd^	81.80 ± 9.20^b^	15.90 ± 5.33^bc^
RS6	2.47 ± 0.64^c^	80.71 ± 6.12^b^	27.13 ± 6.19^b^
GO20	21.4 ± 2.35^b^	NT	
ZO4	4.13 ± 1.06^c^	NT	

*N.B.:*Values are mean ± standard deviation. Within each column, values followed by the same lowercase letter are not significantly different according to Duncan’s multiple range test (*p* = 0.05)

*NT* Not tested, as strains didnot show a promising control activity in vitro

According to Table 8, from an initial OLS infestation estimated to ≥ 80% on leaves, final rates of infestation on old leaves after three months of treatments were as follows: 98.20%, 75.10% and 71.80% for the negative control (DSW), the positive control (BM) and JS7 strain, respectively. On young leaves, decreases of infestation rates were more important: 64.20%, 21.40% and 17.90% for the negative control (DSW), the positive control (BM) and JS7 strain, respectively.

### Blue mold postharvest biocontrol assay

**Fig. 8:**
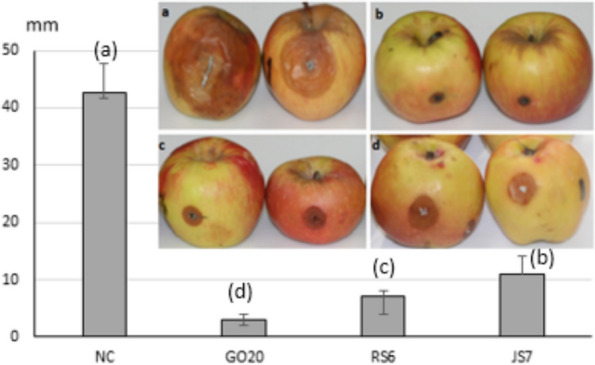
Effect of *Bacillus* strains treatments on diameters of lesion zones caused by *P. expansum* after one week in room temperature comparing to the negative control (NC): a- NC, b- strain GO20, c- strain RS6, d- strain JS7. Lowercase letters between brackets indicates homogenous groups based on Duncan’s multiple range test (*p* = 0.05)

The antifungal activity of *Bacillus* strains against *P. expansum* on apple fruits was expressed by lesion diameters (Fig. 8). After one week at room temperature, sizes of blue mold lesions were significantly reduced in treated fruits (GO20 = 3 mm; RS6 = 7 mm and JS7 = 11 mm) as compared to the negative control (NC = 42.66 mm).

## Discussion

Soil and rhizosphere environments are rich in *Bacillus* species with known biocontrol and plant growth-promoting properties [50]. Saline ecosystems such as Mediterranean Sabkhas have recently been explored as promising sources of beneficial microbes [1, 12] and biocontrol [4, 13].

Therefore, Tunisian Sabkhas have been targeted for mining antimicrobial activity in *Bacillus* communities for the biocontrol of economic important plant diseases in the Mediterranean. Four isolatesJS7, RS6, GO20 and ZO4 have shown a broad-spectrum antagonistic activity in vitro that is likely attributed to several genes involved in the expression of different antimicrobial compounds. The four strains were identified as *B. velezensis*but subdivided into two different genetic groups according to if they were recovered from soil (JS7 and ZO4) or rhizosphere (RS6 and GO20). Promising results were obtained using these strains for the biocontrol of particularly crown gall, olive leaf spot and blue mold diseases in vivo.

Out of a total of 192 bacterial isolates examined, 66 putative *Bacillus* strains were screened for their biocontrol activities. DLM, DCA and AWD assays were efficient in detecting potential biocontrol agents against a variety of bacterial and fungal plant diseases. The antimicrobial activity was detected in strains from soil as much as from rhizosphere. The differences in diameter of inhibition zones recorded between DCA and AWD is likely due to different biocontrol mechanisms that can be deployed in dual cultures such as competitions for nutrient and space, whereas only antibiosis can be detected in AWD. One *Bacillus* strain from each of the four Sabkhas biotopes wasselected for more detailed investigation. This selection of strains JS7, RS6, GO20, and ZO4was based on a combined evaluation of broad-spectrum antimicrobial activities and inhibition zone diameters. Besides antagonistic activities, the local *Bacillus* strains have shown promising thermotolerant enzyme activities such as proteinase and chitinase, which may be used for industrial applications. Preliminary characterization of these compounds has shown that they were of a protein nature and thermotolerant (up to 60 °C), which may be useful in producing commercial enzymes. These findings support the assumption that micro-organisms from saline environments have many enzymatic activities of interest [60, 65].

The characterization of the antimicrobial activity of *Bacillus* strainsdemonstrated that they encode the biosynthesis of a wide range of bioactive compounds, which makes these strains potent antagonists against a wide variety of microorganisms, such as it has been suggested that bacteria of the genus *Bacillus* play an important role in the fight against plant diseases thanks to their various products (lipopeptides, polyketides and bacteriocins) [9, 52]. In our study, the relatively greater retention of antimicrobial activity after thermal treatment observed in JS7 and GO20 suggests these strains produce thermostable bioactive compounds, which could be advantageous for practical field use under high temperature or fluctuating environmental conditions. Similar observations have been reported for other *B. velezensis* strains: for example, surfactin isoforms from *B. velezensis* SK retained full antimicrobial activity when heated up to 80 °C. [5]. Also, in *B. velezensis* Y12, antibacterial compounds remained active over the wide temperature range of 4 90 °C [34].

The genomes of *Bacillus* strains appear to harbor the genes responsible for the biosynthesis of lipopeptides which have potent antagonistic activity against a wide variety of microorganisms particularly the surfactin and iturin families which exhibit antibacterial activity and the fengycins show antimicrobial activity against filamentous fungi in particular [9, 43, 49]. For example, surfactins exert antimicrobial activity against *Salmonella thyphimurium, M. luteus* and *S. aureus* [14], against *E. coli* and on the spores of *B. cereus* [27],

These lipopeptides are known to act synergistically as suggested by several studies on surfactin with iturin [35], surfactin with fengycin [44], iturin with fengycin [56] and iturin with surfactin and fengycin [19], as is the case of the surfactin and iturin reduced infection caused by *Salmonella enteridis* [26], *Pseudomonas syringae* [3], and that by *Pectobacteriumcarotovorum* [71].

Besides antimicrobial activity, lipopeptides have been implicated in the attachment of plant surfaces to the formation of biofilms and the induction of resistance to plant pathogens [44].

It is also possible that the broad-spectrum antibacterial activity observed in the different strains is due to its significant production of the polyketides difficidin, bacillaene, macrolactin, and bacilysin, as suggested by Compaoré et al. [19]. Difficidin had an antibacterial effect against *S. aureus, Clostridium perfringens, Clostridium difficile, S.thyphimurium, E. coli* and others [72], while bacillaene was bacteriostatic against, e.g. *B. thuringiensis, E. coli* and *S. aureus* [47].

Previously, the species *B. subtilis* was the best known for the synthesis of active compounds.In recent years, however, several works have revealed that the related species *B. velezensis*is able to produceadiversified range of powerful bioactive compounds holding promising antimicrobial activities for agricultural applications [46].

Molecular-taxonomic identification revealed that the four (for this study) selected Bacillus strains from Tunisian Sabkhasbelong to two sub-groups of the species *Bacillus velezensis:* one group contains strains RS6 and GO20 which are capable of producing bacteriocins; subtilin (spaS) and ericin (eriC, eriSa and eriSb). In contrast, strains JS7 and ZO4 form adistinct group thatdoes not carry the subtilin and ericin biosynthesis gene.The genetic divergence between rhizospheric and telluric *B. velezensis* strains, particularly in their bacteriocin gene profiles, may reflect ecological adaptation. This divergence could influence not only their antagonistic activity spectrum but also their interaction with host plants. Such ecological differentiation is significant, as rhizospheric environments exert strong selective pressures favoring microorganisms with enhanced antimicrobial capabilities to compete for resources and foster beneficial plant–microbe interactions [18, 38]. Consequently, the presence of bacteriocin-producing genes in rhizospheric strains RS6 and GO20 may represent an adaptation to this competitive niche.

Biocontrol assays in vivo have demonstrated promising results for the use of these strains in the management of crown gall on almond, olive leaf spot disease and blue mold postharvest on apple. While strains JS7 and RS6 showed the greatest control of crown gall and olive leaf spot, strain GO20 was the best in reducing blue mold symptoms. Although strain ZO4 has respectable antimicrobial activity in vitro, mainly against *A. fabrum* C58, it didn’t show the same performance of disease control in vivo. Effectively, pot assays showed a clear-cut difference between the efficacy of strains in vitro and *in planta*. Such lack of correlation between in vitro result and biocontrol in vivo has been documented in other studies with *Bacillus* spp. and further bacterial species such as*Pseudomonas fluorescens* [50, 51, 54, 69]. Much of the inconsistency in the performance of antagonistic bacteria has been attributed to variability in the physical and chemical properties within the ecological niches occupied by biocontrol agents and by the host, which affect both colonization and expression of biocontrol mechanisms [58].

## Conclusion

*Bacillus velezensis* strains isolated from saline soils in Tunisia have been tested and characterized for their antagonistic activities towards bacterial and fungal plant pathogens. Their genomes have shown an important diversity of genes involved in lipopeptide biosynthesis such as iturins, surfactin, fengycin, polyketides and dipeptides. The synthesis of such bioactive compounds makes these *B. velezensis* strains promising biocontrol candidates for many plant diseases. Actually, biocontrol in vivo assays of crown gall, olive leaf spot and blue mold postharvest have demonstrated the efficacy of these strains in reducing disease symptoms. Nevertheless, the evaluation of their action in disease management at larger demo-sites and feasibility studies of their application—such as mass-production, commercialization, and life cycle assessment—are still required for the implementation of their usage in sustainable agriculture.

## Supplementary Information

Supplementary Material 1

Supplementary Material 2

## Data Availability

Sequence data generated in this study are publicly available from the Open Science Framework (OSF) database at the following link: https://osf.io/tn2sc/wiki?wiki=jctqg, and were also deposited in GenBank with accession numbers PQ583503-PQ583514 and PQ623110-PQ623113. All data analyzed during this study are included in the manuscript. However, the phylogenetic analysis of the 16S rRNA region can be available from the corresponding author upon request.

## Abbreviations

AP: Proteinase activity
AWD: Agar well diffusion method
CFU: Colony-forming unit
DCA: Dual-culture assay for fungi
DLM: Double layer method
DNS: Dinitrosalicylic Acid Reagent
GNL: GéloseNutrimentLaitier
LBA: Luria Bertani Agar
MG: Mannitol-Glutamatebroth
ML: Maximum Likelihood
MLSA: Multilocus Sequence Analysis
NAG: N-acetyl glucosamine
NAG: N-acetyl glucosamine
NJ: Neighbor Joining
OLE: Olive leaf extract
PCR: Polymerase Chain Reaction
PDA: Potato Dextrose Agar
PGP: Plant growth promotion
PKs: Polyketide compounds
SDW: Sterile distilled water
YPG: Yeast Peptone Glucose
