# Supplementary material for: Bacillus strains from Tunisian Sabkhas as promising biocontrol agents for several plant diseases in the Mediterranean
Source: BMC Microbiol. 2026 Mar 25;26:323. doi: 10.1186/s12866-026-04819-w (PMC13064316; doi:10.1186/s12866-026-04819-w)
Supplement: Supplementary file 1 — Supplementary Material 1 [file 12866_2026_4819_MOESM1_ESM.docx]

**Additional material**

**Supp. Table 1.** PCR primers used in this study.

|  |  | **Gene Acronym** | **Primer Designation** | **Primer Sequence (5’ =>3’)** | **Reference** |
| --- | --- | --- | --- | --- | --- |
| Molecular characterization: bacteriocines/ lipopeptides/ polycétides | Subtilosin | ywiB  sboA  albA | Osbo P1N  Osbo P2N | CCTCATGACCAGGACTTCGCCTT  CGGTGCCGAGCGCTTCAGGT | Kaboré et al., 2012 |
|  | Ericin | eriC  eriSa | Eric_F  Eric_R | TCAACTGACCGGGCAGGAGC  AAGTATTTGGCCTACAGCGACTCG |  |
|  | Subtilin | spaS | SpaS_Fwd  SpaS_Rev | CAAAGTTCGATGATTTCGATTTGGATGT  GCAGTTACAAGTTAGTGTTTGAAGGAA | Sutyak et al., 2008 |
|  | Mycosubtilin | myc/itu | Am1-F  Tm1-R | CAKCARGTSAAAATYCGMGG  CCDASATCAAARAADTTATC | Tapi et al., 2010 |
|  | Fengycin | fen | Af2-F  Tf1-R | GAATAYMTCGGMCGTMTKGA  GCTTTWADKGAATSBCCGCC |  |
|  | Plipastatin | pps | Ap1-F  Tp1-R | AGMCAGCKSGCMASATCMCC  GCKATWWTGAARRCCGGCGG |  |
|  | Surfactin | srf/lch | As1-F  Ts2-R | CGCGGMTACCGVATYGAGC  ATBCCTTTBTWDGAATGTCCGCC |  |
|  |  | srfA | SRFA-F1  SRFA-R1 | AGAGCACATTGAGCGTTACAAA  CAGCATCTCGTTCAACTTTCAC | Chung et al., 2008 |
|  |  | sfP | SFP-F1  SFP-R1 | ATGAAGATTTACGGAATTTA  TTATAAAAGCTCTTCGTACG |  |
|  | Sublancin | sunT | SUNT-F1  SUNT-R1 | GCTTTGTTAGAAGGGGAGGAAT  CTTGTCCCAACCCATAGGATAA |  |
|  | Iturin | ituD | ITUD-F1  ITUD-R1 | TTGAAYGTCAGYGCSCCTTT  TGCGMAAATAATGGSGTCGT |  |
|  |  | ituC | ITUC-F1  ITUC-R1 | CCCCCTCGGTCAAGTGAATA  TTGGTTAAGCCCTGATGCTC |  |
|  | Iturin A | ituA | ITUD1F  ITUD1R | GATGCGATCTCCTTGGATGT  ATCGTCATGTGCTGCTTGAG | Sarangi et al., 2009 |
|  | Bacillaene | baeA | baeR_F  baeR_R | ATGTCAGCTCAGTTTCCGCA  GATCGCCGTCTTCAATTGCC | Compaoré et al., 2013 |
|  | Macrolactin | mnlA | mlnA_F  mlnA_R | CCGTGATCGGACTGGATGAG  CATCGCACCTGCCAAATACG |  |
|  | Bacilysin | bacA/B  bac B | bacA/B_F  bacA/B_R | TGCTCTGTTATAGCGCGGAG  GTCATCGTATCCCACCCGTC |  |
|  | Bacillomycin | bmyA | bmyA_F  bmyA_R | CTCATTGCTGCCGCTCAATC  CCGAATCTACGAGGGGAACG |  |
|  | Difficidin | dfnA | dfnA_F  dfnA_R | GGATTCAGGAGGGCATACCG  ATTGATTAAACGCGCCGAGC |  |
| Molecular Identification: 16S/MLSA | | 16S | fD1  rP2 | AGAGTTTGATCCTGGCTCAG  ACGGCTACCTTGTTACGACTT | Weisburg et al., 1991 |
|  |  |  | 27F  1492R | AGAGTTTGATCCTGGCTCAG  GGTTACCTTGTTACGACTT | Rooney et al. (2009) |
|  |  | *gyrA* | gyrA-42f  gyrA-1066r | CAGTCAGGAAATGCGTACGTCCTT  CAAGGTAATGCTCCAGGCATTGCT |  |
|  |  | *rpoB* | rpoB-2292f  rpoB-3354r | GACGTGGGATGGCTACAACT  ATTGTCGCCTTTAACGATGG |  |
|  |  | *purH* | purH-70f  purH-1013r | ACAGAGCTTGGCGTTGAAGT  GCTTCTTGGCTGAATGAAGG |  |
|  |  | *groEL* | groEL-550f  groEL-1497r | GAGCTTGAAGTKGTTGAAGG  TGAGCGTGTWACTTTTGTWG |  |
