# Supplementary material for: Bacillus strains from Tunisian Sabkhas as promising biocontrol agents for several plant diseases in the Mediterranean
Source: BMC Microbiol. 2026 Mar 25;26:323. doi: 10.1186/s12866-026-04819-w (PMC13064316; doi:10.1186/s12866-026-04819-w)
Supplement: Supplementary file 2 — Supplementary Material 2 [file 12866_2026_4819_MOESM2_ESM.docx]

**Additional material**

**Supp. Table 2.** - *In vitro* ass

essment of the antimicrobial activity of putative *Bacillus* strains isolated from Tunisian Sabkhas against phytopathogenic strains according to the Double Layer Method (DLM) and the Dual Culture Assay (DCA).

|  |  | Double Layer Method (DLM)  (inhibition zone in mm) | | | | | Dual Culture Assay (DCA) (inhibition in %) | | | | |
| --- | --- | --- | --- | --- | --- | --- | --- | --- | --- | --- | --- |
|  | Isolate codes | *A. tumefaciens* C58 | *A. vitis* (CFBP5523) | *X. campestrispv. juglandis* (1325.2b) | *X. campestris* (339.6) | *P. carotovorum* (1001) | *Verticillium dahliae* V4 | *Rhizoctonia bataticola* | *Fusarium solani* | *Fusarium oxysporum* | *Penicillium expansum* |
|  |  |  |  |  |  |  |  |  |  |  |  |
| Sebkhet de Sidi El Heni, El Djem, Sousse, Soil | JS1 | 0 | 0 | 0 | 0 | 0 | 0 | 0 | 0 | 0 | 0 |
|  | JS2 | 2,9 | 2,6 | 0 | 0 | 0 | 20 | 33,5 | 34,65 | 30,1 | 0 |
|  | JS3 | 0 | 0 | 0 | 0 | 0 | 0 | 21,6 | 0 | 0 | 0 |
|  | JS4 | 0 | 0 | 0 | 0 | 0 | 11,3 | 13,5 | 37 | 41 | 12 |
|  | JS5 | 0 | 0 | 0 | 0 | 0 | 0 | 0 | 0 | 0 | 0 |
|  | JS6 | 0 | 0 | 0 | 0 | 0 | 0 | 0 | 0 | 0 | 0 |
|  | JS7* | 22.96 ± 0.24 | 13.52 ± 0.27 | 18.03 ± 1.35 | 9.98 ± 0.42 | 6.7 ± 0.35 | 68.17 ± 0.66 | 33.12 ± 0.95 | 61.17 ± 0.64 | 46.67 ± 0.31 | 48.50 ± 0.41 |
|  | JS8 | 0 | 0 | 0 | 0 | 0 | 0 | 0 | 0 | 0 | 0 |
|  | JS9 | 6,1 | 5,6 | 6,5 | 2,1 | 0 | 14,6 | 40,6 | 33,3 | 33 | 13 |
|  | JS10 | 0 | 0 | 0 | 0 | 0 | 0 | 0 | 0 | 0 | 0 |
|  | JS11 | 0 | 0 | 0 | 0 | 0 | 0 | 0 | 0 | 0 | 0 |
|  | JS12 | 0 | 0 | 0 | 0 | 0 | 0 | 0 | 0 | 0 | 0 |
|  | JS13 | 0 | 0 | 0 | 0 | 0 | 0 | 0 | 0 | 0 | 0 |
| El Ataya, Kerkennah, Sfax, Rhizosphere of Zigophyllum album | RS1 | 11,8 | 11 | 5,2 | 1,1 | 1,2 | 56,3 | 46,7 | 38 | 41 | 51 |
|  | RS2 | 0 | 0 | 0 | 0 | 0 | 0 | 10,5 | 0 | 0 | 7,33 |
|  | RS3 | 0 | 0 | 0 | 0 | 0 | 0 | 0 | 0 | 0 | 0 |
|  | RS4 | 0 | 0 | 0 | 0 | 0 | 0 | 0 | 0 | 0 | 0 |
|  | RS5 | 0 | 0 | 0 | 0 | 0 | 0 | 0 | 0 | 0 | 0 |
|  | RS6* | 19.58 ± 0.15 | 11.92 ± 0.68 | 11.12 ± 0.4 | 8.23 ± 0.6 | 5.64 ± 0.17 | 56.87 ± 1.52 | 22.12 ± 0.74 | 66.13 ± 0.47 | 41.77 ± 0.14 | 57.38 ± 0.33 |
|  | RS7 | 2,8 | 3,03 | 0 | 0 | 0 | 23 | 19,6 | 20 | 18 | 24,3 |
|  | RS8 | 0 | 0 | 0 | 0 | 0 | 0 | 0 | 0 | 0 | NT |
|  | RS9 | 0 | 0 | 0 | 0 | 0 | 0 | 0 | 0 | 0 | NT |
|  | RS10 | 0 | 0 | 0 | 0 | 0 | 0 | 0 | 0 | 0 | 0 |
|  | RS11 | 11,58 | 11,87 | 0 | 0 | 3,7 | 10,5 | 14 | 16,5 | 33 | NT |
|  | RS12 | 0 | 0 | 0 | 0 | 0 | 0 | 0 | 0 | 0 | NT |
|  | RS13 | 0 | 0 | 0 | 0 | 0 | 0 | 0 | 0 | 0 | NT |
|  | RS14 | 0 | 0 | 0 | 0 | 0 | 0 | 0 | 0 | 0 | NT |
|  | RS15 | 3,1 | 2,4 | 0 | 0 | 1,4 | 0 | 10 | 11 | 0 | 0 |
|  | RS16 | 6,24 | 2,4 | 0 | 2,3 | 3,7 | 27 | 33 | 31 | 12 | 25 |
|  | RS17 | 0 | 0 | 0 | 0 | 0 | 0 | 0 | 0 | 0 | 0 |
|  | RS18 | 0 | 0 | 0 | 0 | 0 | 0 | 0 | 0 | 0 | 0 |
|  | RS19 | 0 | 0 | 0 | 0 | 0 | 0 | 0 | 0 | 0 | 0 |
|  | RS20 | 0 | 0 | 0 | 0 | 0 | 0 | 0 | 0 | 0 | 0 |
|  | RS21 | 0 | 0 | 0 | 0 | 0 | 0 | 0 | 0 | 0 | 0 |
| Chott el Fejej, Rejim, Rhizosphere of *Phoenix dactylifera L.* | GO1 | 0 | 0 | 0 | 0 | 0 | 0 | 0 | 0 | 0 | NT |
|  | GO2 | 0 | 0 | 0 | 0 | 0 | 0 | 0 | 0 | 0 | 0 |
|  | GO3 | 0 | 0 | 0 | 0 | 0 | 0 | 0 | 0 | 0 | NT |
|  | GO4 | 3,9 | 2,3 | 0 | 0 | 0 | 0 | 0 | 0 | 0 | 0 |
|  | GO5 | 0 | 0 | 0 | 0 | 0 | 0 | 0 | 0 | 0 | 0 |
|  | GO6 | 0 | 0 | 0 | 0 | 0 | 0 | 0 | 0 | 0 | 0 |
|  | GO7 | 0 | 0 | 0 | 0 | 0 | 0 | 0 | 0 | 0 | NT |
|  | GO8 | 6,58 | 5 | 0 | 0 | 0 | 12 | 23 | NT | NT | NT |
|  | GO9 | 0 | 0 | 0 | 0 | 0 | 0 | NT | 0 | NT | NT |
|  | GO10 | 0 | 0 | 0 | 0 | 0 | 0 | 0 | NT | 0 | NT |
|  | GO11 | 0 | 0 | 0 | 0 | 0 | NT | 0 | NT | NT | NT |
|  | GO12 | 0 | 0 | 0 | 0 | 0 | NT | 0 | NT | NT | NT |
|  | GO13 | 0 | 0 | 0 | 0 | 0 | NT | 0 | NT | NT | NT |
|  | GO14 | 0 | 0 | 0 | 0 | 0 | NT | 0 | 0 | NT | NT |
|  | GO15 | 0 | 0 | 0 | 0 | 0 | NT | 0 | NT | 0 | NT |
|  | GO16 | 0 | 0 | 0 | 0 | 0 | NT | 0 | 0 | NT | NT |
|  | GO18 | 6,24 | 0 | 1,2 | 0 | 0 | NT | 0 | 0 | NT | NT |
|  | GO20* | 12.03 ± 0.08 | NT | 13.36 ± 0.56 | NT | NT | 62.88 ± 1.66 | 12.96 ± 0.66 | 70.18 ± 0.61 | 42.16 ± 0.70 | 58.33 ± 0.26 |
| Chott el Fejej, Rejim Maatoug, Gabes, Soil | ZO1 | 10,3 | NT | 0 | NT | 2,4 | 0 | NT | 0 | NT | 0 |
|  | ZO2 | 0 | 0 | 0 | 0 | 0 | 0 | NT | 0 | NT | 0 |
|  | ZO3 | 0 | 0 | 0 | 0 | 0 | 0 | NT | 0 | NT | 0 |
|  | ZO4* | 17.65 ± 0.37 | NT | 10.13 ± 0.41 | NT | 6.42 ± 0.15 | 61.63 ± 0.77 | 32.61 ± 0.73 | 52.61 ± 0.64 | 43.18 ± 0.82 | 0 |
|  | ZO5 | 0 | NT | 0 | NT | 0 | 0 | NT | 0 | NT | 0 |
|  | ZO6 | 0 | NT | 0 | NT | 0 | 0 | NT | NT | NT | NT |
|  | ZO7 | 0 | NT | 0 | NT | 0 | 0 | NT | NT | NT | 0 |
|  | ZO8 | 0 | NT | 0 | NT | 0 | 0 | NT | 0 | NT | 0 |
|  | ZO9 | 0 | NT | 0 | NT | 0 | 0 | NT | 0 | NT | 0 |
|  | ZO10 | 0 | NT | 0 | NT | 0 | 0 | NT | 0 | NT | NT |
|  | ZO11 | 0 | NT | 0 | NT | 0 | 0 | NT | 0 | NT | 0 |
|  | ZO12 | 0 | NT | 0 | NT | 0 | 0 | NT | 0 | NT | NT |
|  | ZO13 | 0 | NT | 0 | NT | 0 | 0 | NT | 0 | NT | 0 |
|  | ZO14 | 5,18 | NT | 0 | NT | 2,3 | 0 | 0 | 0 | 0 | 0 |

NT: not tested

(*) selected strains for further analysis and for which DLM and DCA were carried in triplicate.
